# Factors influencing intermittent preventive treatment for malaria prevention among pregnant women accessing antenatal care in selected primary health care facilities of Bwari Area Council, Abuja Nigeria

**DOI:** 10.1371/journal.pone.0277877

**Published:** 2022-12-15

**Authors:** Grace Olufunke Peters, Mergan Naidoo

**Affiliations:** Discipline of Family Medicine, School of Nursing and Public Health, University of KwaZulu-Natal, Durban, South-Africa; University of Washington, UNITED STATES

## Abstract

**Background:**

Although studies in Nigeria showed the efficacy of intermittent preventive treatment using sulfadoxine-pyrimethamine (IPT-SP) in preventing malaria in pregnancy among Nigerian women there is still poor implementation of the intervention in Nigeria.

**Methods:**

A mixed method study was conducted in Bwari Area Council, Nigeria in 2018. The quantitative part of the study is presented and discussed in this paper. Pregnant women were interviewed using a validated interviewer-administered questionnaire and observations of current practice were performed.

**Results:**

A total of 422 pregnant women were recruited into the study (mean age, 26 years) with the majority being married women (90.3%). Most respondents (68.5%) did not know who could take IPT-SP and 58.5% of respondents did not know when and how many times IPT-SP should be taken during pregnancy. Nearly all participants (99.5%) did not take SP at the facility under direct observation of the health worker. None of the facilities had free SP and all respondents paid for SP through the Drug Revolving Fund. The knowledge of the use of SP was significantly influenced by respondents’ parity, ward of residence, antenatal clinic (ANC) attendance history and education. Respondents who had tertiary and secondary education were 8.3 (95% CI: 1.01–68.27) times more likely to use IPT-SP than those without formal education.

**Conclusion:**

Most women who attend ANC in Bwari Area council did not receive IPT-SP as per the national guidelines. The unavailability of logistics (SP, Water and Cup) on a regular basis, the cost of the SP, poor knowledge of the importance of IPT in malaria prevention, and the non-implementation of the administration of SP under direct observation were factors influencing the use of IPT-SP. Outcomes could be enhanced through the provision of measures to address identified gaps by this study.

## Introduction

Malaria continues to be a major public health challenge facing pregnant women in sub-Saharan Africa [SSA] [1, 2]. The high maternal mortality and morbidity, including low birth weight, preterm delivery, severe anaemia, especially in women in their first pregnancy, are associated with malaria in pregnancy (MiP). Documented evidence confirms up to 10,000 maternal deaths due to MiP annually [1, 3, 4]. Three-quarters of the world’s malaria occurs in India and ten sub-Saharan African countries (Burkina Faso, Cameroon, the Democratic Republic of the Congo, Ghana, Mali, Mozambique, Niger, Nigeria, Uganda and the United Republic of Tanzania) [5–9].

The efforts and investment of the Roll Back Malaria (RBM) partnership and other development partners in malaria prevention are justified due to the adverse effects of malaria on pregnant women and their unborn children. To tackle the challenge of MiP in regions with high transmission, the World health organization (WHO) recommends a three-pronged approach [10–13]. These include effective case management of malaria infection, the use of insecticide-treated nets (ITN) [14] and intermittent preventive treatment using sulphadoxine-pyrimethamine (IPT-SP) [10, 15–18]. These are meant to reduce the burden of MiP [12].

The use of IPT consists of administration of a prophylactic dose of an efficacious antimalarial drug, Sulphadoxine-Pyrimethamine(SP), at least thrice during the second and third trimesters of pregnancy to pregnant women by healthcare workers(HCWs) under direct observation treatment(DOT) [15]. SP is given at four weeks intervals and can be given at the later stages of pregnancy without the concerns of side effects [19]. This occurs during routinely scheduled antenatal clinic (ANC) visits. This dosage guideline was updated in October 2012 by the WHO, which stated that at each scheduled ANC contact from the second trimester (13th week), SP could be given monthly until the delivery period without the fear of adverse effects. The WHO in 2016 compiled guidelines on antenatal care for a positive pregnancy experience. One of the recommendations is a minimum of eight ANC contacts for pregnant women, which will allow pregnant women to access three or more doses of SP until delivery [20]. This improves the previously recommended four ANC visits of the focused antenatal care (FANC) model [12].

IPT-SP should be administered from the fourth month of pregnancy, every month until delivery [17, 21–23], during routinely scheduled ANC visits regardless of whether the woman is infected [10, 24, 25]. The drug is dispensed under supervision during ANC visits. SP is the drug currently recommended for the IPT strategy [11, 26–28]. The SP has a good safety profile and remains a good option for IPT in endemic areas in Africa [29–31]. The best place of contacting pregnant women for IPT is the ANCs. Studies show that 60–70% of women attend ANC at least once during any pregnancy in Nigeria [11, 12, 27, 28, 32].

Despite several strategies and programmes to mitigate the impact of MiP, initiated by the Government of Nigeria, development partners, and funding organizations, Nigeria still has a long way to go in achieving targets set for IPT-SP [33, 34]. Although studies in Nigeria showed the efficacy of IPT-SP in preventing anaemia in pregnancy among Nigerian women, there is still low coverage of the intervention in Nigeria. This study assessed IPT-SP use among pregnant women attending ANC services and determining factors influencing IPT-SP use in selected primary health care (PHC) facilities of the Bwari area council (BWAC).

## Methods

### Study design

We conducted a mixed-method study to assess factors influencing the use of IPT-SP by pregnant women in selected PHC facilities of BWAC, one of six area councils of Abuja that enjoys moderate climatic conditions year-round. The data from the qualitative study has already been published [35], and this study will focus on the quantitative data. Five (Dutse, Igu, Kuduru, Usahfa and Bwari) out of the ten wards of BWAC were selected for the study. Two (Ushafa and Igu) of the five wards were rural, and the remaining three(Dutse, Kuduru and Bwari) were urban. The study was conducted in eight facilities that provide ANC services in the five[(Dutse: Dutse Alhaji PHC); (Igu: Tokulo PHC); (Kuduru: Sabon Gari PHC&Kuduru PHC); (Ushafa: Jigo PHC &Ushafa PHC); and (Bwari: Barangoni PHC &Kogo PHC)] selected wards of the BWAC over six months (A ward is an administrative area under a council/local government area that consists of an average of ten villages and each village consists of an average of 100 households).

Consenting pregnant women in the second or third trimesters who were 18 years of age or older attending the selected PHC facilities for ANC were included in the study. The sample size was drawn from the population of pregnant women accessing ANC in the selected PHC facilities. This was based on a stratified sampling technique. Using the Cochran approach, we used the formula for calculating sample size for a mixed-method study. The total sample size (n) was calculated using the following formula.


$$n=\frac{z^{2}pq}{d^{2}}$$

Where n = estimated sample size (when population is greater than 10,000), z = level of statistical significance at 95% confidence level i.e. = 1.96.; p = 48% (48%, *i*.*e*., national prevalence of malaria in pregnancy according to the Federal Ministry of Health) = 0.48; q = 1-p = 0.52 (52%); d = allowable margin of error = 5% = 0.05.


$$n=\frac{1.96^{2}\times 0.48\times 0.52}{0.05^{2}}=383.545344$$

The calculated sample size was 384, and to cater for non-response, attrition, and possible loss of questionnaires, an additional 10% was included to give a final sample size of 422 participants. Since the number of deliveries varies for different PHC facilities, a proportionate method was used to determine each facility’s sample size. The sample for each facility was calculated by weighting the total sample size required with the relative proportion of clients (total deliveries for each facility, as reported by the heads of the facilities) for the week before data collection as numerator; and the sum of deliveries in all the facilities as denominator. The sample sizes for the eight facilities are as follows: Barangoni = 9, Kogo = 41, Dutse Alhaji = 242, Tokulo = 5, Kuduru = 5, Sabon Gari = 57, Jigo = 15, Ushafa = 48. Quantitative data was collected using a structured pre-tested interviewer-administered questionnaire (S1 File) translated into the Hausa language for respondents who could not speak/understand English. The questionnaire contained seven sections, socio-demographic factors, and knowledge of IPT-SP, attitude towards malaria and the use of IPT-SP at ANC, the practice of IPT-SP at ANC, ITN use, antenatal record information, and staff attitudes. Data collection was done by six trained field workers and the principal investigator. The team were trained on quantitative data collection using interviewer-administered questionnaires. The focus was on factors influencing knowledge and attitude of IPT-SP use practice.

The principal investigator developed the interviewer-administered questionnaire based on study objectives and questionnaire used on similar previous study. It also adopted a checklist used for a similar study. Before data collection, the instruments were discussed, validated, and adopted by the research team. The validation of the tool was done during research team review meetings where the researchers’ role played, discussed, and understood the meaning of each of the questions in the data collection tool. Each research assistant was given five copies of the pre-test questionnaire, and responses were shared and agreed upon. After that, data collection was conducted on agreed clinic days (Tuesdays & Thursdays) from January 24 –May 9 2018.

### Measurements

#### Variables

The outcome variable was the use or non use of IPT-SP in index pregnancy. A total of 11 independent variables were analyzed in the bivariate analysis. Among them were knowledge of IPT-SP (how, why, and when to start the use IPT-SP, the dosage and number of times to use IPT-SP in pregnancy), as means to prevent malaria in index pregnancy. Other independent variables were obstetric and socio-demographic characteristics of the respondents such gestational age at registration for ANC in index pregnancy, gestational age at the time of interview, parity/number of live birth, education, age, marital status, tribe, religion, place of residence, and major source of income.

The knowledge and attitude (KA) scale was developed and used to assess pregnant women’s level of knowledge and attitude regarding the use of SP for IPT. A Knowledge, Attitude and Practices (KAP) survey is a quantitative method (predefined questions formatted in standardized questionnaires) that provides access to quantitative information on a specific target population [36]. The scale was based on eleven questions that were designed to assess the knowledge of pregnant women for timing and correct use of SP for IPT.

One point was awarded for a correct answer and zero points for a wrong answer. The KA score was evaluated by counting participants’ correct responses for each knowledge and attitude question (this makes our overall KA score). The score was aggregated, and the mean score was computed to determine the overall KA score of participants; participants with a score greater than, equal to and less than the mean scores were grouped to have good, fair and poor KA to the use practice of SP for malaria prevention respectively. The explanatory variables were, age, marital status, educational level (tertiary, secondary, primary/vocational, none), tribe, religion, ward/place of residence (rural, urban), major source of income (employed, owned business, unemployed), gestation age at registration (1^st^ trimester, 2^nd^ trimester, 3^rd^ trimester), ANC attendance history(once, twice, thrice), and parity (none, one, two, three, four).

### Analysis

The data obtained for the quantitative part were cleaned, coded and entered into a Microsoft Excel file. The data were summarized to describe factors supporting or inhibiting the use of malaria prevention drugs among pregnant women by demographic stratification. The data analysis was conducted using SPSS version 26. Continuous variables were presented as means and standard deviation (SD), while categorical variables were presented as percentages. A scoring system was adopted to grade the overall knowledge and attitude toward IPT usage among respondents. Correctly answer IPT usage related questions was scored “1”, while the incorrect response was graded “0”. The overall knowledge scores were divided into thirds with the lowest third grade poor and highest graded good. On the other hand, overall attitude was divided into two with the highest termed positive attitude.

Bivariate and multivariate analyses were conducted to determine the influence of some explanatory variables (age, education, antenatal visits, and parity) on SP usage (outcome variable). The selection of the explanatory variables for the logistic regression was based on study objectives, previous literature and findings from the bivariate analysis. The comparison for the outcome variable, SP usage, was based on the previous history of taking at least one dose of SP. Chi-square was used (where appropriate) to compare proportions at a 5% significance level, and relationships were tested using a logistic regression model.

Ethical approvals for this study were obtained from Federal Territory Health Research Committee, Abuja Nigeria (Ref No: FHREC/2017/01/84/30-10-2017), Bwari Area Council Primary Health Care Department and University of Kwazulu-Natal Biomedical Research Ethics Committee (BREC Ref No: BE557/17). Before the commencement of data collection. Participants’ privacy (non-disclosure of identity) was agreed upon and written informed consent was obtained from all participants.

## Results

### Socio-demographic and obstetric characteristics of the respondents

**Fig 1:**
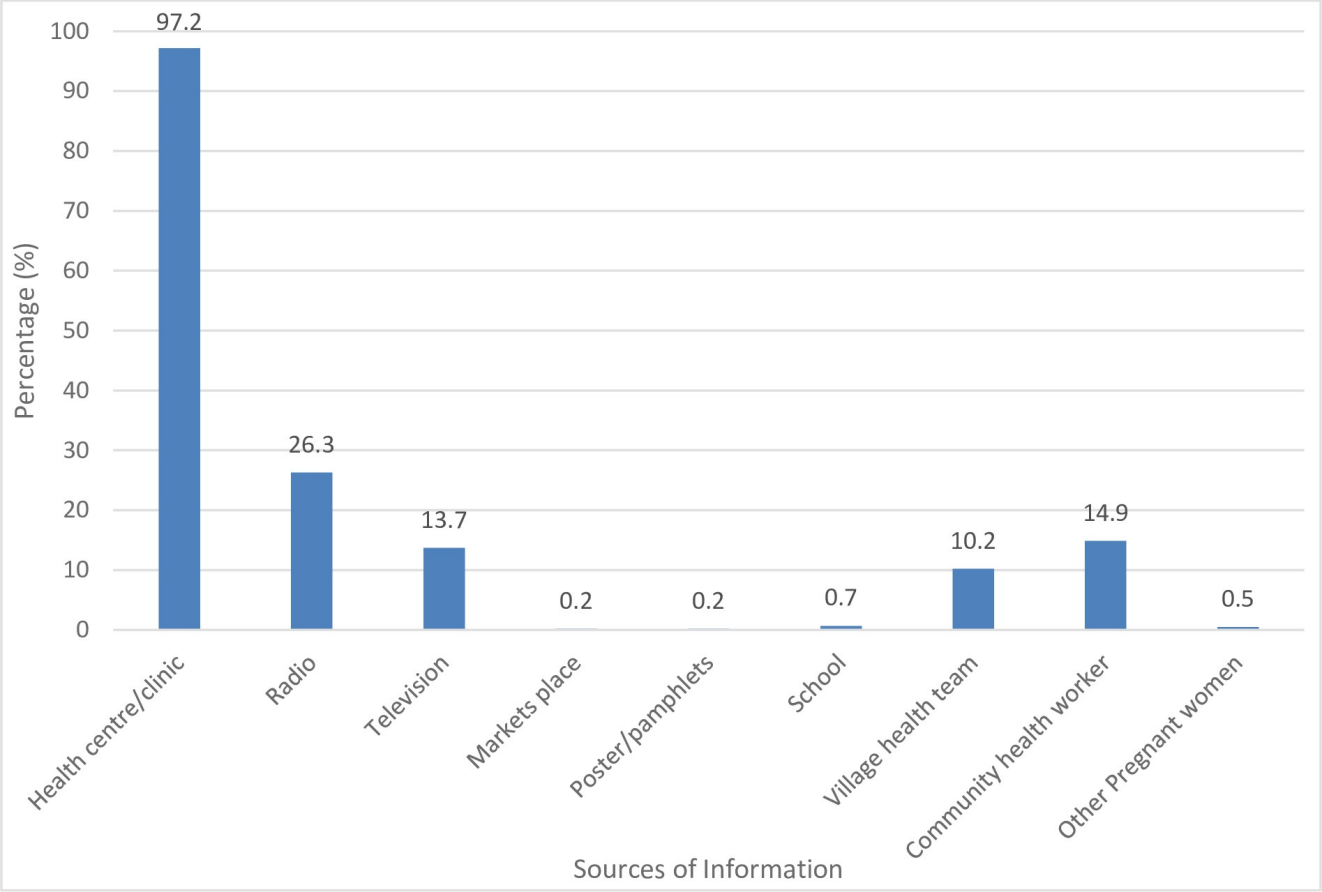
Sources of IPT^a^ information.

Four hundred and twenty-two pregnant women attending ANC in the selected PHC facilities participated in the study. The socio-demographic characteristics of the respondents are summarized in Table 1. Most (97.2%) claimed to have learnt about IPT from the health facilities (Fig 1) during health talks given by HCWs on their ANC days.

**Table 1:** Socio-demographic and obstetric characteristics of pregnant women attending ANC in BWAC, Nigeria.

Characteristics	Frequency, n = 422 (%)
Age	
< 20	60 (14.2)
20-35^a^	343 (81.3)
36 and above	19 (4.5)
Mean age 26 years SD (5.3) Range 18–41	
Marital Status	
Married	381 (90.3)
Single (widowed, co-habiting)	41 (9.7)
Education- Level	
Tertiary	102 (24.2)
Secondary (senior and junior)^c^	270 (63.9)
Primary/vocational	43 (10.2)
None	7 (1.7)
Tribe	
Gbagyi	264 (62.6)
Hausa	92 (21.8)
Yoruba	27 (6.4)
Ibo	29 (6.9)
Others	10 (2.4)
Religion	
Christianity	306 (72.7)
Islam	115 (27.3)
Ward/Place of residence	
rural	68(16.1)
urban	354(83.9)
Major source of income	
employed	100 (23.7)
owned business	196(46.4)
unemployed	126(29.9)
Gestational age at registration for ANC	
First trimester	4 (0.9)
Second trimester	411 (97.4)
Third trimester	7 (1.7)
Gestational age at the time of interview	
Second trimester	157(37.2)
Third trimester	265 (62.8)
Number of previous live births	
None	101 (23.9)
One	159 (37.7)
Two	130 (30.4)
Three	28 (6.6)
Four	4 (1)
Number of SP doses taken	
1	84(19.9)
2	136(32.2)
≥3	202(47.9)
ANC visit history	
1	47 (11.1)
2	154 (36.5)
3	186 (44.1)
≥ 4	35 (8.3)

Most women obtained information on IPT from the health facility. Women received information from more than one source in some instances.

### Knowledge and attitude towards the use practice of IPT-SP

About 93.6% of the respondents knew that malaria could kill; however, their knowledge of other debilitating effects such as anaemia (35.1%), spontaneous abortion (31.8%), intrauterine death (32.7%), low birth weight (40.5%), and prematurity (31.0%) were poor. Overall, 23.7% of the respondents had good knowledge of intermittent preventive treatment of malaria, and the majority (69.0%) of them had fair knowledge about IPT, while 7.3% had poor. The majority (95.5%) displayed a positive attitude towards using IPT, while 4.5% had a negative attitude (Table 2).

**Table 2:** Knowledge, attitude and intermittent preventive practice (IPT).

Knowledge Category	Correct responses (%)
Knowledge of malaria severity*	
Malaria can: Kill	395 (93.6)
Cause anaemia	148 (35.1)
Cause spontaneous abortion -	131 (31.8)
Cause intrauterine death	138 (32.7)
Cause low birth weight	171 (40.5)
Cause prematurity	131 (31.0)
Knowledge Malaria Prevention and Treatment*	
I can prevent malaria by: Wearing long-sleeved clothes	330 (78.2)
Making fire and smoke	18 (4.3)
Spraying insecticide	408 (96.7)
Trimming and cutting bushes around my house and environment	416 (98.6)
Taking herbal preparations	19 (4.5)
Using mosquito repellent	332 (78.7)
Using insecticide-treated nets	353 (83.6)
Using a malaria prevention drug	274 (64.9)
Knowledge of when SP start	172 (40.7)
Number of tablets (SP)	380 (90.1)
Number of times SP can be taken in pregnancy	232(54.9)
Pregnant woman who cannot take SP	127(30.3)
There are no safety concerns about IPT use during pregnancy	104 (24.6)
Practice	
Direct observed use of SP by a health worker	
No	420(99.5)
Yes	2 (0.5)
Take home SP	
Yes	305 (72.3)
No	117 (27.6)
ANC attendance (absent)	
Yes	3 (0.7)
No	419 (99.3)
Overall knowledge of IPT use	
Good	100 (23.7)
Fair	291(69.0)
Poor	31(7.3)
Overall attitude towards IPT use	
Positive	403(95.5)
Negative	19(4.5)

Note: a scoring system was used to grade the overall knowledge and attitude of respondents to IPT use.

### Satisfaction on antenatal care services and IPT-SP administration

The exit interview revealed that less than half (48.8%) of the respondents claimed that malaria in pregnancy was explained. Only about a quarter (25.1%) confirmed ways of treating and preventing malaria, while 34.4% confirmed that health workers spoke about malaria prevention drugs (SP). A little over one fifth (21.6%) claimed they were taught how to use the prescribed medication (Table 3).

**Table 3:** Exit interview-: Satisfaction on ANC/IPT services.

Services rendered during ANC	Freq. n = 422 (%)
Paid and spent some money (SP, tests, etc)	421 (99.8)
General Health talk given	421 (99.8)
Likely pregnancy problems discussed	353 (83.6)
Malaria in pregnancy included	206 (48.8)
Malaria Prevention and treatment discussed	106 (25.1)
Malaria prevention drug(SP) introduced	145 (34.4)
SP available	377 (89.3)
How to use SP taught	91 (21.6)
Likely adverse reaction and what to do taught	4 (0.9)
Took SP under direct observation at the clinic	-
Prompt and timely services rendered (attention given)	407 (96.4)
Privacy guaranteed service	419 (99.3)
Skilful HCWs at clinic	418 (99.1)
Well manneredHCW(attitude)	418 (99.1)
Satisfactory service	413 (97.9)

The bivariate analysis (chi-square test) showed utilization of SP was not statistically significantly associated with maternal age, gestational age at booking, marital status, tribe religion or major source of income. However, the factors found to be associated with use of Intermittent Preventive Treatment (IPT-SP) were: education (χ2 = 12.67; p = 0.005), and type of place of residence (χ2 = 14.378; p = < 0.0001). These findings are depicted in Table 4.

**Table 4:** Socio-demographic characteristics of participants according to the uptake of IPT-SP.

Factors	IPT Usage^a^	Non-usage	P-value
	Frequency (%)	Frequency (%)	
Age			0.469
<20	54(13.8)	6(13.3)	
*20–35*	320(81.8)	23(74.2)	
*36 and above*	17(4.4)	2(6.5)	
Marital status			0.502
*Married*	355(90.8)	26(83.9)	
*Single*	36(9.2)	5(16.1)	
Educational level			0.005*
*Tertiary*	94(24.0)	8(25.8)	
*Secondary*	255(65.2)	15(48.4)	
*Primary/ vocational*	37(9.5)	6(19.4)	
*None*	5(1.3)	2(6.4)	
Tribe			0.869
*Gbagyi*	249(63.7)	15(48.4)	
*Hausa*	87(22.3)	5(16.1)	
*Yoruba*	24(6.2)	3(9.7)	
*Igbo*	23(5.8)	6(19.4)	
*Others*	8(2.0)	2(6.4)	
Religion			0.92
*Christianity*	247(63.2)	26(83.9)	
*Islam*	144(36.8)	5(16.1)	
Ward/Place of residence			<0.0001*
*Rural*	56(14.3)	12(38.7)	
*Urban*	335(85.7)	19(61.3)	
Major source of income			0.379
*Employed*	94(24.1)	6(19.4)	
*Owned business*	180(46.0)	16(51.6)	
*Unemployed*	117(29.9)	9(29.0)	
Gestational age at registration			0.122
*1*^*st*^ *trimester*	3(0.7)	1(3.2)	
*2nd trimester*	383(98.0)	29(93.6)	
*3*^*rd*^ *trimester*	5(1.3)	1(3.2)	
Gestational age at the time of interview			0.348
*2nd trimester*	148(37.9)	9(29.0)	
*3*^*rd*^ *trimester*	243(62.1)	22(71.0)	
ANC attendance history			0.004*
*Once*	45(11.5)	2(6.5)	
*Twice*	146 (37.3)	8(25.8)	
*Thrice*	170 (43.5)	16(51.6)	
≥4	30(7.7)	5(16.1)	
Parity/number of live birth			0.027*
*None*	92(23.5)	9(29.0)	
*One*	137(35.0)	5(16.1)	
*Two*	131(33.5)	12(38.8)	
*Three*	27(6.9)	5(16.1)	
*Four*	4(1.0)	0()	

* = Statistically significant at 95% CI

*Education, place of residence, number of doses taken

^a^ = had history IPT-SP usage atleast once

The relationship between the educational level and IPT-SP usage showed that the majority (89.2%) of those that had a previous history of IPT-SP usage had a minimum secondary educational level. Also, a similar proportion (85.7%) of those with a history of IPT-SP usage resides in Urban while a noticeable percentage (38.7%) of those without a history of usageresides in rural areas. Usage of IPT-SP was common among those who had a history of two and three antenatal visits and those with one or two histories of live births.

### Relationship between facilitating factors practice of SP use and socio demographic factors among pregnant ANC users

The logistic regression analysis results showing the relationship between the participant’s SP use and their socio-demographic factors is shown in Table 5. The level of education, parity, ward of residence, and ANC attendance history of the participants influence their odds of having good knowledge of SP use. Participants with primary education are 12.816 (p<0.05) times more likely to have good knowledge of SP use than those with no formal education, which is the reference group. On the other hand, the participants with tertiary and secondary education are 95.055 (p<0.05) and 9.185 times respectively more likely to have good knowledge of SP use than those with no formal education, after controlling for the effect of co-founders using multivariate analysis. Participants’ education, ward of residence, and antenatal visits significantly affect their odds of having good knowledge of SP usage.

**Table 5:** Factors influencing the use of IPT-SP among pregnant women attending ANC at BWC, Abuja, Nigeria.

Factors	P-value	Odds ratios	95% C.I. for EXP(B)	
			Lower	Upper
Age	0.867	0.986	0.841	1.157
Parity	0.025*	0.834	0.366	1.898
Marital status (married)	0.352	0.312	0.027	3.622
Education	0.026			
tertiary	0.003**	95.055	4.727	1911.587
secondary	0.033*	9.185	1.19	70.872
primary/vocational	0.048*	12.816	1.027	159.883
Tribe	0.998			
Gbayi	0.920	0.875	0.063	12.055
Hausa	0.960	0.927	0.048	18.008
Yoruba	0.977	1.049	0.041	27.082
Igbo	0.880	1.289	0.048	34.899
Religion (Christianity)	0.906	1.073	0.333	3.461
Residence (rural)	0.001**	0.165	0.058	0.474
Antenatal visits	0.019*	0.784	0.436	1.413
Source of income				
Employed	0.713	1.322	0.299	5.835
Owned business	0.269	1.906	0.607	5.984
Gestation age at reg	0.355	1.092	0.906	1.316
Constant	0.615	4.693		

** means significant at 1% Confidence level

* means significant at 5% level. Outcome variable = history of IPT usage (at least once).

## Discussion

This study aimed at assessing factors that influence the use of IPT-SP among pregnant women attending ANC in PHC facilities of BWAC. Respondents’ use of SP according to national guidelines, the WHO model of administration, and recommended doses for IPT-SP was very poor compared to the RBM benchmark target for all pregnant women in areas with the moderate-to-high transmission in Africa [7]. Maternal age, gestational age at the first ANC visit, marital status, and religion have no influence on SP use among participants in this study.

In this study, the age range of 20–35 years constituted most of the participants compared with other age groups and most registered in the second trimester. This is not surprising because the age range is the peak reproductive age for women. The second trimester during which most of the respondents initiated ANC is acknowledged by the WHO as the expected gestational age to start using IPT [9, 14, 24, 25]. This allows participants an opportunity to attend ANC at least three times before delivery. From a study in Kenya, married women were more likely to use SP than the unmarried women [37]. Married women may get financial and psychosocial support from their spouses to attend the ANC clinic to receive adequate IPT-SP. A study in the Bungoma East district of Kenya confirmed that women who received support from their partners during antenatal care were 8.2 times more likely to take subsequent IPT-SP doses after the first one [38].

Studies conducted in the Ekiti State of Nigeria [3] and Bukoba, Tanzania [39] confirms early ANC booking positive association with the use of IPT-SP. The more pregnant women attend the ANC, the higher the exposure toward health information on IPT-SP, hence the higher likelihood of receiving optimal doses of SP. The findings are in line with the studies conducted in Tanzania [40, 41], Burkina Faso [42], Ghana [43–45], Mali [46], Benin [47], Cameroon [48] and Malawi [49]. ANC visits provide an ideal opportunity to promote IPT-SP [50]. The more visits a pregnant woman makes to the ANC, the higher the number of SP doses she would receive, as long as the visits are scheduled at least one month apart. Our study findings confirm that pregnant women’s continuing use of IPT-SP depends on their regular ANC attendance [29].

It was observed in this study that, participants area of residency where they attend ANC care impact their use of SP significantly. Residing or attending ANC in urban areas avail pregnant women easy access to the health facility and access to logistics such as pharmacies as an alternative means of obtaining medication during drug stock outs [35]. Facilities within urban areas are accessible and staffed better than those in rural areas [51]. Women who attended rural health facilities were less likely to complete the recommended SP doses during pregnancy. This finding is similar to a study done in Geita district, Tanzania [26] and elsewhere in Nigeria [51]. The rural health facility often suffers from poor stock levels of SP, poor supply chain from the district health office due to challenges in transportation and understaffing [4]. This leads to high client to-staff ratios and long queues and waiting times, leading to some pregnant women defaulting their ANC visits [52]. Our finding that parity significantly improves the likelihood of using ANC services and the use of IPT-SP possibly related to prior delivery experiences differs from other studies. However, studies conducted in Nigeria [52] Uganda [53] contradict these findings, where parity did not affect the use of SP.

In this study, having attained education status above secondary level positively influenced the use of SP by the respondents. This finding is not unexpected as the majority of the medium used in malaria prevention campaigns is more accessible to educated people among the population. It could also be due to their ability to read and understand drug prescriptions in the absence of DOT and health experts. The result of this report is similar to the findings of Exavery and his colleagues [41]. They state that improved education attainment considerably influenced antimalarial drugs usage during pregnancy. Similarly, the findings from other studies in Nigeria [52], Malawi [54, 55], Ghana [56], and Zimbabwe [57] showed the knowledge on the SP and on the consequences of not taking IPT-SP as a facilitator toward the uptake hence the association between education level and the likelihood of the uptake of three or more doses of SP for malaria prevention during pregnancy.

The analysis from five African countries has shown that the facilities having IPT guidelines and implementing IPT as part of their routine ANC services significantly improved IPT delivery [31]. This study further revealed that pregnant women did not take their SP under direct observation of the health workers. There was general non- implementation of direct observed use of SP in all facilities surveyed. They were allowed to go home with their SP. This was in line with findings from some studies [3, 13] in some other parts of Nigeria where compliance to direct observation use of IPT-SP was poor or minimal.

A similar study conducted by Mubyazi and his colleagues [58] observed that uptake of SP was low among the study participants especially when women were allowed to take the SP at home. In this study, attending ANC without eating breakfast was a key reason given by health workers for non-compliance to DOT [35] and confirmed by pregnant women. Another similar study in Malawi by Yoder and colleagues [59] negate this finding of non-compliance; where nurses complied with direct observed use of SP. This was an affirmation to pregnant women reliance on health workers. This can positively influence uptake of SP by direct observation. If it is utilized as demonstrated by a study conducted in the Gambia [60], where findings showed that women relied entirely on health workers to provide safe drugs, at the correct time. These were similar to findings from a study in Tanzania [40] that reported facility and policy factors as serious impediments to IPT-SP coverage. It is established that availability and adherence is critical to improving the use of SP and implementation of DOT scheme [61].

This knowledge gap may also be due to lack of personal and institutional updates on new interventions in preventing malaria during pregnancy in the district or even from the gap in number of staffs who might not have enough time to explain to all the pregnant women during ANC visit(s). Participants not taking the SP at the facility under direct observation of the HCWs were "a missed opportunity [61]”.

The factors responsible for this behaviour were not restricted to pregnant women alone. Studies had shown that not only the pregnant women but healthcare workers and the healthcare system related issues play a major role in the use or non use of SP by pregnant women [61]. Some other studies confirm poor mastery of SP guidelines and confusion about whether SP can be given on an empty stomach among healthcare providers [3, 40, 51, 53, 59, 61–64]. This confusion stemmed from a combination of unclear policy and guidance, inadequate training, poor knowledge and attitudes, insufficient supportive supervision, and lack of information and job aids on IPT [64, 65]. Several studies reported conflicting national policies on IPT provision when treatment for malaria was needed [66]. Some healthcare providers expressed uncertainty over the effectiveness of SP for IPT. There is an urgent need for health departments to update national IPT policies aligned to the most recent WHO IPT policy and implementation strategies with simplified guidance on IPT dosing. This will serve as an important opportunity for national programmes to update and reinvigorate their IPT strategy [15, 67].

### Recommendations

A major determinant of utilization of IPT among the study population was the knowledge of prophylaxis for malaria prevention. For pregnant women to use IPT-SP properly, they must be well informed about the dangers of malaria related to pregnancy and receive the appropriate prevention and treatment therapy at the right time during pregnancy. There is therefore a need to continuously sensitize and educate pregnant women about the use and benefits of SP. The challenge of including SP among other ANC routine drugs should be addressed. Sensitization programs should be designed to target different age group of pregnant women at the antenatal clinics and in the community. The HCWs should be targeted for training to address the miss opportunity and utilize early attendance of ANC by pregnant women. There should be production of posters and fliers, which should be distributed to pregnant women and well displaced on the walls of ANC clinics like those of nutrition, immunization and other programmes. Various religious places of worship and electronic media such as radio and television should be further encouraged and strengthened. Ways of improving availability of free SP and ITNs should be explored in all facilities to address payment of any form of fee whether subsidized or not among pregnant mothers accessing ANC in PHC facilities.

### Study limitation

One of the limitations of the study was that it took place in only one area council of Abuja, the Federal Capital Territory with a total of six area councils. Secondly, the interview took place on clinic days within the premises of the facilities. A number of techniques were employed to improve trustworthiness and achieve a comprehensive understanding of the findings: data from a variety of participants and different sources (interviews and observations) were triangulated; as well as verification of information in records and health registers.

## Conclusion

In conclusion, most women who attend ANC in BWAC did not receive IPT-SP as per the national guidelines. The cost of the SP, poor knowledge of the importance of IPT in malaria prevention, and the non-implementation of the administration of SP under direct observation were factors influencing the use of IPT-SP. Therefore, positive outcomes on the use of SP could be enhanced through the provision of measures to address identified gaps by this study.

## Supporting information

S1 File Questionnaire for ANC clients (pregnant women). (DOCX)

S2 File Checklist for ANC facility observation and health workers DOT performance. (DOCX)

S1 Table (XLSX)

S2 Table (XLSX)

## Abbreviations

ANC: Antenatal Clinic
BWAC: Bwari Area Council
DOT: Direct Observation Treatment
FANC: Focus Antenatal Care
FCT: Federal Capital Territory
HCWs: Health Care Workers
IPT: Intermittent preventive treatment
IEC: Information, education and communication
IPT-SP: Intermittent preventive treatment with Sulphadoxine Pyrimethamine
ITN: Insecticide treated net
KAP: Knowledge, attitude and practice
MiP: Malaria in pregnancy
MoH: Ministry of Health
NDHS: Nigeria demographic and health survey
NPC: National Population Commission
NWGs: National Working Groups
PHC: Primary Health Care
SEM: Socio-Ecological Model
SP: Sulphadoxine Pyrimethamine
SSA: Sub-Sahara Africa
WHO: World Health Organisation
