# Supplementary material for: Factors influencing intermittent preventive treatment for malaria prevention among pregnant women accessing antenatal care in selected primary health care facilities of Bwari Area Council, Abuja Nigeria
Source: PLoS One. 2022 Dec 15;17(12):e0277877. doi: 10.1371/journal.pone.0277877 (PMC9754266; doi:10.1371/journal.pone.0277877)
Supplement: S1 File — (DOCX) [file pone.0277877.s001.docx]

**QUESTIONNAIRE FOR ANC CLIENTS (Pregnant women)**

Date …………………Ward…………………………….. ANC Clinic/Health facility……………………………………………………………………

Questionnaire Code No:

**SECTION1: SOCIO-DEMOGRAPHIC FACTORS**.

Age:………………………. Place of residence: …………………………

Gravidity: Parity:……………… No of live births:………………………….

Gestational age at first visit……………………………………………………

No. of weeks gestation:………………………………………………………….

1. Marital Status: Married ( ) Widowed ( ) Single ( ) Co-habiting ( )
2. Highest level of education: Tertiary ( ) Senior Secondary School ( ) Junior Secondary School ( ) Primary ( ) Technical/Vocational ( ) None ( ) Other, specify…………………………
3. Tribe : Gbagyi () Hausa () Yoruba () Ibo() Others, specify…………………………….
4. Language of communication: Gbagyi () Hausa () Yoruba () Ibo() English () Others, specify…………….
5. Does any member of your household own electronic equipment (e.g. TV, radio, cassette, etc.) at present? (Tick only one box)
   1. No ( )
   2. Yes ( )
6. Do you or any member of your household have at least one handset? (Tick only one box)
   1. No ( )
   2. Yes ( )
7. What is your main source of income? (Tick only one)
8. Formal employment (e.g. nurse, teacher, secretary, accountant, etc.)  (1)
9. Trading, commerce, selling (e.g. wholesalers, retailers, petty traders, etc.) (2)
10. Agriculture, livestock, forestry, fisheries (e.g. subsistence farmers, market vendors, etc.)  (3)
11. Craft/creative workers (e.g. tailor, hairdresser, building, wood trades, metal and machinery) (4)
12. Casual or wage labour (construction workers, farm labourers, etc.) (5)
13. Support from friends/family (husband, remittance from friends/family)  (6)
14. Support from institutions (government, NGO payments, pensions, etc.)  (7)

**Section 2: KNOWLEDGE OF IPT-SP**

4 . Have you ever heard about Malaria?

1. Yes ()
2. No ()

5 . Which vector transmit malaria to humans? (Tick one only)

1. Rat ()
2. Dog ()
3. Mosquito ()
4. Fly ()
5. Cockroach ()
6. I don’t know

6 . Malaria can be transmitted to human by?

1. Drinking contaminated water ()
2. Eating contaminated food ()
3. Bite of mosquito infected with Malaria ()
4. Coming into close contact with malaria patients ()
5. I don’t know

7 . What do you think are the most common signs of malaria of malaria infection? (Tick all that apply)

1. High temperature/ Fever ()
2. Loss of energy
3. Vomiting
4. Sweating
5. Headache
6. Body pains
7. Itching
8. Loss of appetite
9. Chills
10. Dizziness
11. I don’t know
12. Others

8. Malaria is a maternal, newborn and child health issue because these groups of people are most at risk for infection.

1. True ( )
2. False ( )
3. I don’t know

9. Malaria poses a serious health risk to the pregnant woman, but the unborn child is protected by the placenta from the harmful effects of malaria.

1. True ( )
2. False ( )
3. I don’t know

10 . The World Health Organization recommends the following interventions for malaria in pregnancy in areas where malaria is common (moderate –to- high transmission) (circle all that apply)

1. Use insecticide – treated nets (ITNs) ( )
2. Monthly preventive (intermittent)treatment (IPT)(fansidar)
3. Indoor residual spraying (IRS)
4. Early diagnosis and prompt treatment for those infected with malaria
5. I don’t know

11. Pregnant women should seek services for prevention of malaria:

1. In community
2. Through traditional healers
3. During regularly scheduled antenatal care visits.
4. I don’t know

12. The World Health Organization recommends that pregnant women in areas of moderate-to- high malaria transmission take IPT with SP:

1. At the beginning of pregnancy
2. At least two times during pregnancy, after quickening
3. At each scheduled antenatal care visit, starting the first dose as early as possible during the 2^nd^ trimester of gestation
4. I don’t know

13. Monthly preventive (SP) treatment should not be taken by pregnant women who:

1. Take folic acid
2. Are HIV-positive and taking cotrimoxazole
3. Sleep under ITN
4. I don’t know

14. The last dose of IPT-SP can be administered up to the time of delivery, without safety concerns

1. True ( )
2. False ( )
3. I don’t know ( )

15. What is the source of your knowledge about the above mentioned medicine?

1. Health centre/clinic ( )
2. Radio ( )
3. Television ( )
4. Market place ( )
5. Poster/pamphlets
6. School
7. Church/Mosque
8. Village Health team
9. Community health worker
10. Drug shop/drug hawker
11. Other pregnant women ( )
12. others, specify ……………………………………………

16. How many times during a pregnancy does a woman have to swallow the tablets at

the ANC in Nigeria?

1. Once ( )
2. Twice ( )
3. Thrice ( )
4. > Three times ( )
5. I don’t know ( )

17. Pregnant women exhibit signs and symptoms of malaria more readily than the general population.

1. True ( )
2. False ( )
3. I don’t know ( )

18. Do you think Malaria can kill you if it’s untreated?

1. Yes ()
2. No ()
3. I don’t know ()

19. What are the effects of malaria on the pregnant woman?

1. Can cause anaemia ( )
2. Can cause death ( )
3. Nothing ( )
4. Don’t know ( )
5. Other, specify………………………………………

20 . What are the effects of malaria on the unborn baby? (Tick all that apply)

1. Can cause spontaneous abortion ()
2. Can cause Intra Uterine Death ( )
3. Can cause low birth weight ( )
4. Can cause prematurity ( )
5. Nothing ( )
6. Don’t know ( )
7. Other, specify…………………………..

21. Which of these are ways to prevent and control Malaria? (Tick all that apply)

1. Sleeping in bed nets ()
2. Wearing long sleeved clothes ()
3. Making fire and smoke ()
4. Spraying insecticide ()
5. Trimming and cutting bushes around the house and environment ()
6. I don’t know ()
7. Others ……………………………………

22. What other ways can a pregnant woman prevent herself from getting malaria?

1. Take herbal preparations ( )
2. Sleep under an insecticide treated net ( )
3. Use mosquito repellent ( )
4. Wear protective clothing, especially at night ( )
5. Don’t know ( )
6. Others, specify…………………………………

23. What personal protection measures do you take to guard against Malaria? (Tick all that apply)

1. Use repellents ()
2. Use mosquito coil ()
3. Use insecticide (Raid, boom, etc.) ()
4. Burn cow dung/leaves ()
5. Use mosquito nets ()
6. Use malaria prevention drug
7. Do nothing
8. Others (specify) -------
9. I don’t know

**SECTION 3: ATTITUDE TOWARDS MALARIA AND THE USE OF IPT-SP AT ANC**

| Attitude towards Malaria and Monthly preventive drug(IPT) after first ANC visit/ quickening | Strongly disagree (1) | Disagree (2) | Undecided (3 | Agree (4) | Strongly agree (5) |
| --- | --- | --- | --- | --- | --- |
| 24. I think that Malaria is a serious and life-threatening disease |  |  |  |  |  |
| 25. Malaria can be transmitted from one person to another like the common cold |  |  |  |  |  |
| 26. I think the best way to prevent myself getting Malaria is to avoid getting mosquito bites cold |  |  |  |  |  |
| 27. The first dose of monthly malaria preventive drug is to be taken at my first ANC visit or at quickening (first movement of the foetus felt) |  |  |  |  |  |
| 28. In my opinion IPT-SP must be taken 2-4 times before delivery |  |  |  |  |  |
| 29. The use of IPT-SP is a sure way of protecting myself and unborn child from malaria |  |  |  |  |  |
| 30. I am sure that anyone can get Malaria |  |  |  |  |  |
| 31. In my opinion, only children and pregnant women are at risk of Malaria |  |  |  |  |  |
| 32. I think that one can recover spontaneously from Malaria without any treatment |  |  |  |  |  |
| 33. If someone has got Malaria, people should avoid having close contact with him/her |  |  |  |  |  |
| 34. I think that it is dangerous when Malaria medicine is not taken completely |  |  |  |  |  |
| 35. I can buy anti-Malaria drugs from the drug shop/pharmacy to treat myself when I get Malaria |  |  |  |  |  |
| 36. I think attending ANC will help me to prevent malaria by taking IPT-SP at the clinic |  |  |  |  |  |
| 37. I think that I should go to the health centre/clinic to have my blood tested as soon as I suspect that I have suffered from Malaria |  |  |  |  |  |
| 38. I will seek for advice or treatment when I get Malaria  39. In my opinion, it is very important to check for an expiry date of the drug before taking it |  |  |  |  |  |

**SECTION 4 : PRACTICE OF IPT-SP AT ANC**

40. How many months into your pregnancy did you first attend ANC?

1. 1-3 month ( )
2. 4-6months ( )
3. above 6months ( )

41. If later than the 6months, why did you attend your first ANC at this time?

1. Did not have any problems during the pregnancy ( )
2. Did not have money for transportation ( ) I could not leave my farm work ( )
3. Long distance to the ANC deterred me ( )
4. Wanted pregnancy to show first ( )
5. Wanted pregnancy to be established culturally ( )
6. Attended the herbalist/spiritualist’s clinic first ( )
7. Was being seen by the TBA ( )
8. Attended a Church/Mosque mission ANC ( )

Other, specify…………………………………………………

42 . Did the health worker/nurse give you some medicine to swallow for them to see on the first

visit?

1. Yes ( )
2. No ( )
3. I don’t know ()

43. If no to question 42, have you ever been given any medicines to swallow for the

nurses to see on your subsequent visits?

1. Yes ( )
2. No ( )
3. Don’t know

44. If yes to question 42 or 43, how many tablets were they?

1. One ( )
2. Two ( )
3. Three ( )
4. Four or more ()

45**.** Did the medicine look like this one? (**Please show a sample of SP)**

1. Yes ( )
2. No ( )

46. If no, how did it look like? .............................................................................

47a. How many times during this pregnancy have you swallowed these same tablets at

the ANC?

1. Once ( )
2. Twice ( )
3. Thrice ( )
4. More than three times ( )
5. can’t remember ( )
6. none ()

47b. Do you have any reason not to swallow the malaria preventive drug after the first dose?

1. Yes ()
2. No ()

47c. If answer to 47b is yes, why?

1. I reacted to the malaria preventive drug ()
2. I don’t want to take drug again ()

Others…………………………………………………………………………………………………………………………………….

………………………………………………………………………………………………………………………………………………….

…………………………………………………………………………………………………………………………………………………….

48. Were you served with free, clean water to take the medicine?

1. Yes ( )
2. No ( )

49. If no, how did you get the water to take the medicine?

Fetched water from the tap ( )

Bought water at the health facility ( )

Had my own water ( )

Other, specify …………………………………………………………………….

50. If yes to question 48, were you served in a cup that was commonly used by other

Pregnant women?

1. Yes ( )
2. No ( )
3. other, specify………………………

51. If yes, was this practice of sharing cups acceptable to you?

1. Yes ( )
2. No ( )

52. Do you miss ANC appointments/Clinic days?

1. Yes ()
2. No ()

53. If yes how frequent?

1. Always
2. Sometimes
3. Rarely

**SECTION 5: ITN USE**

54. Do you own an ITN?

1. Yes ( )
2. No ( )

55. Have you been sleeping under an ITN for this pregnancy?

1. Yes ( )
2. No ( )

56. Did you sleep under the net last night?

1. Yes ( )
2. No ( )

57. Exit Interview: We are trying to learn from those of you that use the Antenatal Care/IPT Services so that we can serve our clients better

|  | **YesNo** |
| --- | --- |
| - 1. Did you spend any money here today? | Y N |
| - 1. Do you think the health worker understood your state as explained by you? | Y N |
| - 1. Did the health worker give health talk? | Y N |
| - 1. Did she/he explain the likely problem of pregnancy? | Y N |
| - 1. Explain to you about malaria in pregnancy? | Y N |
| - 1. Did she/he talk about ways of treating/preventing malaria? | Y N |
| - 1. Did she/he talk about IPT/ malaria prevention drugs? | Y N |
| - 1. Were you given the prescribed IPT drugs at the clinic? | Y N |
| - 1. Did the health worker teach you how to use/take the drugs prescribed? | Y N |
| - 1. Did you take the drug at the facility before the health worker? | Y N |
| - 1. Did the health worker tell you what to do in case there is a reaction? | Y N |
| - 1. Do you think you had to wait too long before the health worker started attending to you today? | Y N |
| - 1. Do you think the health worker spent enough time with you? | Y N |
| - 1. Do you think you had enough privacy during the consultation? | Y N |
| - 1. If you had a choice will you prefer health worker that attended to you to be of the same gender with you? | Y N |
| - 1. Are you satisfied with the skill of the health worker that attended to you? | Y N |
| - 1. Are you satisfied with the attitude of the health worker? | Y N |
| - 1. Are you satisfied with the service you received today? | Y N |
| - 1. If you had a choice, would you prefer to go elsewhere? | Y N |
| - 1. Please tell us anything you think we need to know in order to improve the ANC/IPT services, which we have not mentioned here | Y N |

**SECTION 6 : ANTENATAL RECORD INFORMATION**

58. Total number of visits including this one ( )

59. Gestation (weeks) recorded at first visit ( )

60. Number of doses SP recorded (given) ( )

61. SP administration

Dose of SP given at (please tick) visit

given

1st

2nd

3^rd^

61b. Gestational age at first visit

**SECTION 7 : STAFF ATTITUDE**

62 . Did the staff usually greet you before starting the ANCs?

1. Yes ( )
2. No ( )

63. Did the staff usually smile at you during your ANC visits?

1. Yes ( )
2. No ( )

64. Were you ever shouted at during any of your ANC visits?

1. Yes ( )
2. No ( )

65. Did you feel comfortable to ask the staff questions during your ANC visits?

1. Yes ( )
2. No ( )

66. Were your questions answered to your satisfaction?

1. Yes ( )
2. No ( )

67. Do you feel the staff spent enough time with you during your visits?

1. Yes ( )
2. No ( )

68. Did the way you were treated encourage you to attend the ANC again?

1. Yes ( )
2. No ( )

69. Did the way you were treated encourage you to tell others to attend ANC?

1. Yes ( )
2. No ( )

……………………………………………………………………………………………

……………………………………………………………………………………………

T*hank you very much for your time. This information is very helpful for us. And thank you for choosing to Use ANC/IPT-SP.*
