# Supplementary material for: Factors influencing intermittent preventive treatment for malaria prevention among pregnant women accessing antenatal care in selected primary health care facilities of Bwari Area Council, Abuja Nigeria
Source: PLoS One. 2022 Dec 15;17(12):e0277877. doi: 10.1371/journal.pone.0277877 (PMC9754266; doi:10.1371/journal.pone.0277877)
Supplement: S2 File — (DOCX) [file pone.0277877.s002.docx]

**CHECK LIST FOR ANC FACILITY OBSERVATION AND HEALTH WORKERS DOT PERFORMANCE**

Code number: …………………….Date:………………………………………………….

Name of facility ………………………………………………………………………………

Ward ……………………………………………………………………………………………….

1. Health education program drawn for the quarter includes MIP Yes ( ) No ( )
2. Health education program drawn for the quarter includes IPTp Yes ( ) No ( )
3. Health talk given at ANC on day of visit Yes ( ) No ( )
4. Health talk given that day included malaria in pregnancy Yes ( ) No ( )
5. Health talk given that day included IPTp Yes ( ) No ( )
6. Presence of posters of IPTp/MIP on the wall Yes ( ) No ( )
7. Presence of ANC Report Book for daily summaries Yes ( ) No ( )
8. Presence of ANC Monthly Data returns form Yes ( ) No ( )
9. SP available at ANC Yes ( ) No ( )
10. Practice of DOT observed Yes ( ) No ( )
11. SP given is recorded in ANC report Book for daily summaries Yes ( ) No ( )
12. SP given is recorded in ANC book of clients Yes ( ) No ( )
13. Presence of Adverse Event forms for SP Yes ( ) No ( )
14. Presence of free, clean, safe water for DOT Yes ( ) No ( )
15. Presence of safe, clean water for sale for DOT Yes ( ) No ( )
16. Availability of IPTp National protocol Yes ( ) No ( )
17. Availability of IPTp training manual Yes ( ) No ( )
18. Presence of ITNs for distribution to clients Yes ( ) No ( )

Any additional observations made: ……………………………………………………..

……………………………………………………………………………………………

……………………………………………………………………………………………

T*hank you very much for your time. This information is very helpful for us.*
